# Distribution of Nanoparticles in the See-through Medaka (*Oryzias latipes*)

**DOI:** 10.1289/ehp.9209

**Published:** 2006-07-06

**Authors:** Shosaku Kashiwada

**Affiliations:** Research Center for Environmental Chemical Risk, National Institute for Environmental Studies, Tsukuba, Japan

**Keywords:** bioavailability, distribution, environmental condition, medaka, nanoparticles, salinity, toxicity

## Abstract

**Objective:**

Because the environmental fate of manufactured nanoparticles is considered an emerging environmental concern, I used water-suspended fluorescent nanoparticles (solid latex solution) to investigate the distribution of nanoparticles in the eggs and bodies of see-through medaka (*Oryzias latipes*).

**Results:**

Particles 39.4–42,000 nm in diameter were adsorbed to the chorion of medaka eggs and accumulated in the oil droplets; 474-nm particles had the highest bioavailability to eggs. Particles 39.4 nm in diameter shifted into the yolk and gallbladder during embryonic development. Adult medaka accumulated 39.4-nm nanoparticles mainly in the gills and intestine when exposed to a 10-mg/L nanoparticle solution. Nanoparticles were also detected in the brain, testis, liver, and blood. Concentrations of nanoparticles in the blood of male and female medaka were 16.5 and 10.5 ng/mg blood protein, respectively. These results suggest that nanoparticles are capable of penetrating the blood–brain barrier and that they eventually reach the brain. Salinity-dependent acute toxicity was observed in medaka eggs exposed for 24 hr to nanoparticles.

**Conclusion:**

The bioavailability and toxicity of nanoparticles depend on environmental factors and multiple physicochemical properties. Further studies on the toxic effects of nanoparticles used in commercial products and their environmental relevance, are necessary to define the risks and benefit of nanomaterial applications.

Since the discovery of buckyballs (C_60_ fullerenes), carbon nanotubes (CNTs), and quantum dots (QDs), the challenge to develop new methods of product synthesis and apply these new nano-sized materials has advanced in the United States, European Union (EU), Japan, and other countries. Nanomaterials are defined by the U.S. National Nanotechnology Initiatives (2006) as materials that have at least one dimension in a range of roughly 1–100 nm. Nanomaterials have multiple physicochemical properties, including size-dependent electrical conduction, high tensile strength, high elastic limit and heat tolerance, high chemical stability, hydrophobic or hydrophilic properties, high capacities for electric current transportation and hydrogen storage, superconductivity, ultraviolet light-blocking capability, and antimicrobial activity (Shelley 2005). Thus, nanomaterials have become the next generation of materials used in electronic devices, clothes, sunscreens, and cosmetics. Water-soluble fullerenes show site-selective DNA cleavage (Boutorine et al. 1994; Tokuyama et al. 1993;) and inhibition of HIV protease (Friedman et al. 1993). It is believed that in the future fullerenes will be applicable for disease diagnosis, as artificial vectors for transinfection (Nakamura et al. 2000), and for pinpoint drug delivery targeted at specific sites in the body (Nakamura and Isobe 2003). Furthermore, nanoscale iron is expected to be useful in remediation of contaminated soil (Zhang 2003).

Nanomaterials affect biological behavior at the cellular, subcellular, and protein levels (Braydich-Stolle et al. 2005; Colvin 2003; Ding et al. 2005; Donaldson et al. 2001; Jia et al. 2005; Oberdörster et al. 2005; Sayes et al. 2004; Service 2004; Yamawaki and Iwai 2006) because of redox activity. They have extremely large surface area-to-weight ratios (Oberdörster et al. 2005); these large surface areas can be electrically charged on their surfaces, and some have high redox activities (Colvin 2003). Because of these characteristics, research on the adverse human health effects from exposure to nanomaterials and environmental pollution is needed, as is discussion on the environmental risks posed by these new materials. Industries producing nanomaterials are growing rapidly, as well as the numbers and types of products containing these materials (Zhang et al. 2005). Global spending on nanotechnology research and development is approximately US$9 billion per year (Service 2005). The bioavailability and bioactivity of nanomaterials in the environment will eventually occur because of the environmental release from the industries that produce them, the consumer products that contain them, and the waste products that result from both. Nanotechnology will create a new class of environmental damage (Service 2004), but only US$36.5 million per year is currently being spent on studies targeted at understanding the effects of nanoparticles on human health and the environment in the United States and the EU (Service 2005). To date, air pollution resulting from nanomaterials released into the environment has been the main concern because of the high risk of exposure of people working in and living in proximity to nanotechnology industries (Colvin 2003; Nel et al. 2006; Oberdörster et al. 2005). We need more funding for research on the risks of nanomaterials so that we may understand the effects on human health.

Aquatic environments may also be threatened by pollution from nanomaterials. Oberdörster (2004) exposed juvenile large-mouth bass to C_60_ fullerenes and investigated the resulting induced oxidative stress. Her research indicated a trend toward a decrease of glutathione (GSH) in the gills and an increase of lipid peroxidation in the liver. Gills are important in extracting oxygen from ambient water and are priority organs in xenobiotic exposure. It is well known that xenobiotics are taken up by fish mainly through the gills. Redox-active particles encountered by the gills should therefore induce antioxidant enzyme production and consume GSH. Meanwhile, the brain has a blood–brain barrier that prohibits exposure of the brain to xenobiotics. Lipid peroxidation in the brain would be an indicator that nanomaterials have reached this organ, but unfortunately there is no evidence indicating that nanomaterials reach the brain of fish (Oberdörster et al. 2004). Yamago et al. (1995) studied the *in vivo* biological behavior of a ^14^C-labeled water-miscible C_60_ fullerene in rats. Fullerenes administrated orally were easily eliminated in the feces, but those injected intravenously were retained in the body after 1 week. The intravenously injected fullerenes were distributed mainly in the liver (91.7% of dose), and simultaneously some were able to penetrate slightly the blood–brain barrier. Nevertheless, the distribution of nanomaterials is not well known in animals. Nanomaterials induce inflammation on cell surfaces, penetrate cell membranes, and eventually show cytotoxicity (Yamawaki and Iwai 2006). Although the formation of blood clots through inflammation, and atherosclerosis and cardiac stress are believed to be effects of nanomaterials exposure (Oberdörster et al. 2005), they are unconfirmed in humans and wildlife. A laser diffraction particle-size analyzer is effective for detecting different-sized ultrafine particles (particles with an aerodynamic diameter of < 100 nm) in water, but this method is not material specific. Because environmental water includes natural nano-sized particles, no highly selective analytical method for artificial nanomaterials in the environment has been agreed on. However, we need to understand the fate of nanomaterials in organisms, develop a method of environmental health protection, and eventually reach a consensus on the risk of exposure to nanomaterials in environment.

This present investigation focused on the distribution of nanomaterials in the body of the medaka (*Oryzias latipes*) and how these materials reach the organs, eventually exerting their effects. Fluorescence nano-sized monodispersed particles made of latex (polystyrene) were used as models of nano-sized materials (buckyballs, CNTs, QDs) in this study. The small laboratory fish species, medaka, has been used to investigate waste water toxicology (Ma et al. 2005), endocrine disruptors (Scholz et al. 2004), liver carcinogenesis (Liu et al. 2003), germ cell mutagenesis (Shimada et al. 2005), gene mutagenesis (Winn et al. 2005), and developmental and functional genomics (Ju et al. 2005) because of its small body size (3–4 cm in adults), hardy nature (wide temperature and salinity tolerances), and short generation time (2–3 months). Small fish such as medaka and zebrafish have attracted much interest as remarkable animal models for organogenesis and human disease (Garrity et al. 2002) because they have transparent embryos, rapid embryo development, and organs and tissues that are functionally equivalent to those of mammals (Wittbrodt et al. 2002). Recently, a research group established a pigment-free medaka strain termed the “see-through” (ST II) medaka (Wakamatsu et al. 2001). The ST II medaka serves as a vertebrate model with a transparent body throughout its entire life. The main internal organs (heart, spleen, blood vessels, liver, gut, gonads, kidney, brain, spinal cord, ocular lens, air bladder, gallbladder, and gills) are visible to the naked eye or with a simple stereomicroscope. It was expected therefore that the distribution of fluorescent nanoparticles would be detectable through the skin. Using the ST II, I investigated the distribution of water-suspended fluorescent nanoparticles in living medaka. This study contributes to developments of environmental nanotoxicology.

## Materials and Methods

### Test organism

I obtained see-through medaka (*Oryzias latipes*, ST II strain) from medaka broodstock at the National Institute for Environmental Studies (Tsukuba, Japan). From approximately 50 natural color mutants of medaka in the Laboratory of Freshwater Fish Stocks of the Bioscience and Biotechnology Center, Nagoya University (Nagoya, Aichi, Japan), I selected some that showed deficiency in pigmentation. Medaka has four main pigments (melanophore, iridophore, leucophore, and xanthophore). Wakamatsu et al. (2001) genetically removed these pigments from the entire body by crossing selected mutants, thereby creating a transparent fish (Wakamatsu et al. 2001). Breeding groups of ST II medaka were fed brine shrimp nauplii twice daily and maintained under a 16/8-hr light/dark cycle at 26°C. After these groups had spawned fertilized eggs, the females were netted and the external egg clusters were removed by hand from their abdomens (between the anal and pelvic fins). Filaments attaching the eggs were removed by gently rolling the clusters between moistened papers. The eggs were then rinsed and placed in ERM (embryo rearing medium: 1 g NaCl, 0.03 g KCl, 0.04 g CaCl_2_ × 2H_2_O, and 0.163 g MgSO_4_ × 7H_2_O in 1 L ultrapure water, adjusted to pH 7.2 with 1.25% sodium bicarbonate solution and filtered sterilized) (Yamamoto 1939). Fertilized eggs collected daily were incubated at 26°C in ERM until hatched. Posthatch ST II larvae were fed rotifer, *Brachionus urceolaris*, once a day for the first week and then shifted to brine shrimp nauplii twice a day. For this research, eggs of ST II medaka were used immediately after spawning; adult ST II medaka (male and female, posthatch month 5) were also subjected to treatments. Fish and eggs used in this study were treated humanely and with regard for the alleviation of suffering.

### Exposure designs for nano-sized particles

I evaluated four types of nano-sized distribution: *a*) adsorption and/or accumulation of nano-sized particles by medaka eggs and distribution of nanoparticles in posthatch larvae; *b*) particle size–dependent adsorption and/or accumulation by medaka eggs; *c*) effects of salinity on adsorption and/or accumulation of nano-sized particles by medaka eggs and aggregation of nano-sized particles in solution, and *d*) distribution of nano-sized particles in the blood and organs of adult medaka. Monodispersed non-ionized fluorescent polystyrene microspheres were used to estimate the distribution of nano-sized particles in eggs and embryos. Three groups of 15 eggs each of ST II were exposed to 39.4-nm diameter-sized fluorescent particles at 1 mg/L [2.78% solids-latex (polystyrene) solution; Polysciences, Inc., Warrington, PA, USA] in 10 mL ERM for 3 days under a 16/8-hr light/dark cycle at 26°C with gentle rotary shaking. Exposure solutions were renewed daily. Five eggs from each group were sampled on day 1 and rinsed in ERM. Exposed eggs were observed for adsorption and/or accumulation of nanoparticles under a fluorescence dissecting microscope (model MZ FL III; Leica Microsystems, Tokyo, Japan) equipped with a green fluorescence protein filter (excitation wavelength, 480 nm; emission, 510 nm) to detect fluorescence. The eggs were also sliced to 20-μm thickness using a cryostat (model CM 3050S; Leica Microsystems), and the sections were observed by fluorescence dissecting microscopy. On day 3, all other exposed eggs were rinsed and moved into ERM in the absence of nanoparticles, then incubated continuously until hatched under the same conditions described above. Posthatch larvae of ST II medaka were immediately observed with fluorescence-dissecting microscopy to detect accumulated nanoparticles.

To confirm size-dependent adsorption and/or accumulation of nanoparticles by eggs, I used 39.4-nm [2.78% solids–latex (polystyrene) solution, 474-nm (2.5%), 932-nm (2.7%), 18,600-nm (2.65%), or 42,000-nm (2.7%)] fluorescent particles (Polysciences, Inc.). A 1-mg/L solution of the different-sized particles was prepared individually with ERM, and the eggs of ST II were exposed to the respective solutions for 3 days under the same conditions described above. Exposure solutions were renewed daily. After exposure the eggs were rinsed, and the fluorescences in the envelope/yolk area and in the oil droplet area were observed separately under a fluorescence microscope.

The effects of salinity on the adsorption and/or accumulation of nanoparticles by eggs and aggregation of nanoparticles in solution were estimated by using 39.4-nm fluorescent particles and modified ERMs. Three groups of 15 eggs each of ST II were exposed to 30 mg/L nanoparticles in 1×, 5×, 7.5×, 10×, 15×, 20×, or 30× concentrated ERM for 3 days under the same conditions described above. Exposure solutions were renewed daily. The osmotic pressure of each ERM solution was measured with an osmometer (model OM801; Vogel, Giessen, Germany) and these were 33.3, 167, 250, 333, 500, 666, and 1,000 mOsm/L, respectively. After exposure the eggs were rinsed, and the fluorescence of each whole egg was observed. The suspended concentration of nanoparticles in each nanoparticle ERM solution was measured with a fluorescence microplate reader (Safire; Tecan Japan Co., Ltd., Osaka, Japan; excitation wavelength, 480 nm; emission, 510 nm), with the fluorescence nanoparticle solution as the standard, and the optical densities of each solution were measured with a photometer (BioPhotometer; Eppendorf AG, Hamburg, Germany; wavelength, 600 nm).

To estimate the distribution of nanoparticles in medaka organs, I exposed eight male and eight female ST II adults individually to 39.4-nm fluorescent particles at 10 mg/L in 500 mL ERM for 7 days under identical conditions. Exposure solutions were renewed daily. After exposure, ST II adults were rinsed in ERM and anesthetized with ice-cold ERM. The abdominal areas of the anesthetized fish were observed under a fluorescence-dissecting microscope to detect the fluorescing nanoparticles. After these observations, the tail of each medaka was transected, and the blood collected in a glass capillary tube was mixed with 10 μL of 0.1 M phosphate buffer (pH 7.4) in a 1.5-mL microcentrifuge tube. The blood mixed with buffer was sonicated for 3 min, and its fluorescence measured with a fluorescence microplate reader (Tecan Japan Co., Ltd.) to quantify the concentration of fluorescent nanoparticles. The protein concentration of each blood solution was measured according to the Bradford method (Bradford 1976), with bovine serum albumin as the standard. The gills, kidney, liver, intestine, gonads, and brain were extracted and observed under the microscope for fluorescence.

### Quantification of fluorescent nanoparticles in fish

Completely extracted organs were placed on a glass slide and flourescence images immediately captured with the fluorescence microscope (Leica Microsystems) under the conditions described above. All fluorescence images were captured for 200 msec with a digital camera (Leica DC 350FX; Leica Microsystems) attached to the microscope. To identify organs with fluorescence, regular light images were also captured for 100 msec and the two types of images were overlapped. Egg images were captured such that the oil droplets were uppermost. The fluorescence captured was pseudo-colored green using Leica FW 4000 software (version 1.0.3; Leica Microsystems). Regular light was emitted from the bottom of the glass stage, whereas fluorescent light was emitted down from the top of the object, so the fluorescent image was not affected by the object’s shadow. The fluorescent image was subtracted from the variable background using the same software described above. Fluorescence intensity was quantified with Photoshop software (version 5.5; Adobe Systems Inc., San Jose, CA, USA). All data were analyzed statistically by analysis of variance (ANOVA) with Excel 2003 (Microsoft Co., Tokyo, Japan).

## Results

### Adsorption and accumulation of nano-sized particles by medaka eggs

**Figure 1:**
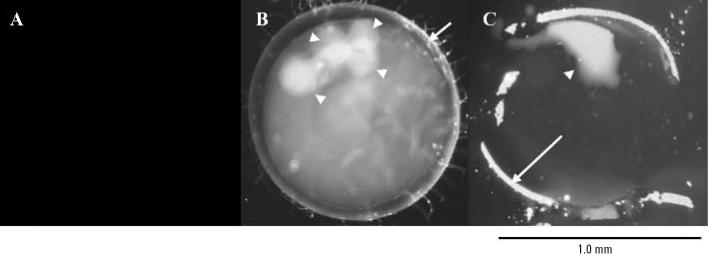
Fluorescence images of fertilized eggs of ST II medaka exposed to 39.4-nm fluorescent nanoparticles in ERM solution (1 mg/L for 24 hr). (*A*) Whole egg image in the absence of nanoparticles. (*B*) Whole egg image in the presence of nanoparticles. Chorion of the egg (short arrow) and oil droplets (arrowheads) show high fluorescence. (*C*) Image of frozen section of exposed egg. Thickness of section was 20 μm. Egg envelope (long arrow) and a coalesced oil droplet (arrowhead) show high fluorescence intensities. Exposure time for a fluorescence image capture was 200 msec.

**Figure 2:**
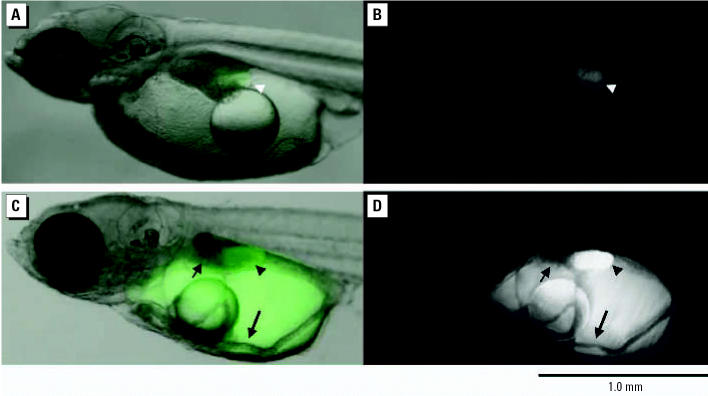
Accumulation of 39.4-nm fluorescent particles in yolk of larval ST II medaka. Fertilized ST II eggs were exposed to 1 mg/L of nanoparticle ERM solution for 3 days, then moved to fresh, clean ERM until hatch. In the control, auto-fluorescence is detected from the gallbladder (white arrowhead) of the larva and slightly from the yolk area: (*A*) overlapped image; (*B*) fluorescence image. In the exposed larva obvious fluorescence is detected from the gallbladder (closed arrowhead) and yolk area: (*C*) overlapped image; (*D*) fluorescence image. Short arrows indicate liver. Long arrows indicate left duct of Cuvier. Exposure time for a fluorescence image capture was 200 msec.

**Figure 3:**
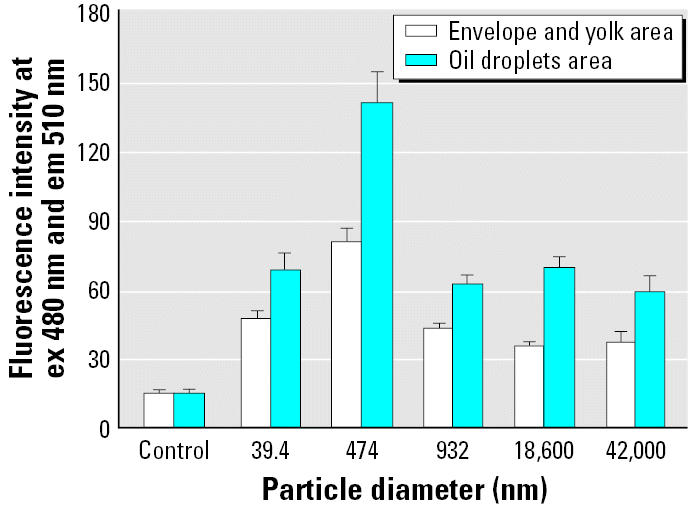
Diameter-dependent distribution of micro-fine fluorescent particles in fertilized eggs of ST II medaka. Three groups of 15 fertilized eggs each were exposed for 3 days to solutions containing particles of different sizes at 1 mg/L in ERM. Exposure time for a fluorescence image capture was 200 msec.

Spawned ST II eggs were exposed to 39.4-nm fluorescent particles made of latex (1 mg/L in ERM). No mortality was observed during the exposure and post-exposure to hatch periods. Fluorescence from the particles was detected in whole eggs (Figure 1B). The egg envelope (chorion) and oil droplets showed higher fluorescence than the yolk area. Examination of frozen sections confirmed that the fluorescent particles had been adsorbed on the chorion and accumulated in the oil droplets (Figure 1C). Examination of the fluorescence images of the posthatch larvae revealed that medaka had intrinsic autofluorescence compounds in the gallbladder (Figure 2A,B). ST II larvae spawned from exposed eggs exhibited highly concentrated fluorescent nanoparticles in the yolk and gallbladder, but no detectable fluorescence was observed from the liver (Figure 2C,D). Fluorescence intensity of the oil droplets area was stronger than that of chorion and yolk areas, which suggested oil droplets have a priority to accumulate latex nanoparticles (Figure 3).

### Particle size–dependent adsorption and accumulation by medaka eggs

Spawned ST II eggs were exposed to 39.4- to 42,000-nm fluorescent particles (1 mg/L in ERM) for 3 days. Fluorescence in the envelope and yolk areas and the oil droplet area was measured separately, although the fluorescence data for the oil droplet area included fluorescence from the envelope wrapped over the oil droplets. Fluorescence was highest in the fish exposed to 474-nm particles and less with smaller and larger particles (Figure 3). The fluorescence intensities of the eggs exposed to 932-, 18,600-, and 42,000-nm particles approximated those of the eggs exposed to 39.4-nm particles. Thus, 474-nm particles were adsorbed easily on, and accumulated by, fertilized medaka eggs.

### Effects of salinity on adsorption and accumulation of nano-sized particles by medaka eggs and aggregation of nano-sized particles in solution

**Figure 4:**
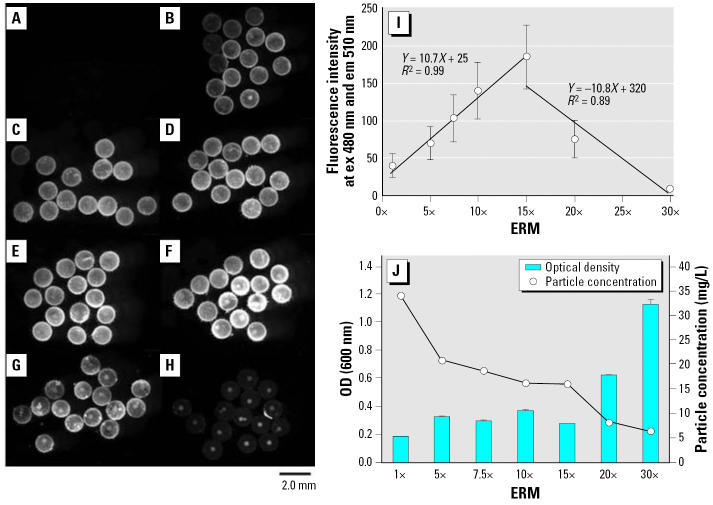
Effects of ERM salinity on adsorption and accumulation by ST II eggs and aggregation of 39.4-nm fluorescent particles in modified ERM solutions. Three groups of 15 eggs each were exposed to each nanoparticle solution for 24 hr. (*A*) Blank, 1× ERM in the absence of nanoparticles. (*B–H*) 1×, 5×, 7.5×, 10×, 15×, 20, and 30× ERM in the presence of nanoparticles at 30 mg/L, respectively. (*I*) Accumulation of fluorescent particles by eggs. Abbreviations: em, emission wavelength; ex, excitation wavelength. Fluorescence value increased linearly with ERM concentration and peaked at 15× ERM, then declined. (*J*) Increase in optical density and decrease in suspended concentration of nanoparticles along with ERM salinity. Aggregations of particles occurred in more concentrated ERM solutions. Exposure time for a fluorescence image capture was 200 msec.

Spawned ST II eggs were exposed to 39.4-nm fluorescent particles at 30 mg/L in 1× to 30× concentrated ERM solutions for 24 hr. The fluorescence of the whole eggs increased linearly and peaked at 15× concentrated ERM, then decreased linearly at higher concentrations of ERM (Figure 4I). Fluorescence in 30× ERM was lower than that in 1× ERM (Figure 4B,H,I). More fluorescence was observed in the oil droplet areas of the eggs compared with the yolk area (Figure 4G,H). The optical density (OD) of the nanoparticle solutions increased irregularly in proportion to the salinity, and simultaneously the suspended concentration of the nanoparticles declined (Figure 4J). This means that aggregation of nanoparticles occurred in the solutions with increasing salinity. Although the suspended concentration of particles was reduced to half at 15× ERM compared with that at 1× ERM, interestingly the adsorption and accumulation peaked at 15× ERM. Furthermore, embryo mortality reached 97.8% at 5× ERM and 100% at 15× ERM (Table 1). Conversely, the 20×- and 30×-ERM solutions exhibited higher ODs but dramatically reduced adsorption and accumulation of particles by eggs compared with the 15× ERM. The aggregations of nanoparticles were observed under the fluorescence microscope (data not shown). This reduction was considered by decrease of suspended concentration of nanoparticles due to the aggregate formation. In the previous exposure to particles at 1 mg/L for 24 hr, there was no lethal toxicity to eggs, but exposure to 30 mg/L for 24 hr had a 35.6% lethal effect on eggs in 1× ERM. Lethal effects increased dramatically with salinity; simultaneously, aggregation occurred and adsorption and accumulation were decreased (Table 1).

### Distribution of nano-sized particles in blood and organs of adult medaka

**Figure 5:**
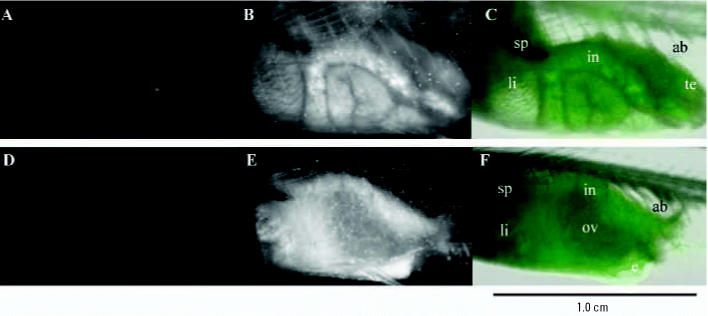
Distributions of 39.4-nm fluorescent particles in adult ST II medaka. Abbreviations: ab, airbladder; e, egg; in, intestine; li, liver; ov, ovary; sp, spleen; te, testis. (*A,D*) Fluorescence images of left abdominal areas of male and female ST II in the absence of nanoparticles. (*B,E*) Fluorescence images of left abdominal areas of male and female ST II exposed to ERM in the presence of nanoparticles (10 mg/L) for 7 days. (*C,F*) Overlapping images of regular light and fluorescence images of male and female to identify organs.

ST II adults were exposed to 39.4-nm fluorescent particles at 10 mg/L for 7 days and observed for the accumulation of particles in the blood and organs. There was no mortality during the exposure. Fluorescence was detected from the liver, intestine, and gonads through the transparent skin of living ST II, but it was not detected from the spleen (Figure 5). Fluorescence was also detected from removed organs [gills, kidney, liver, intestine, ovary, testis, brain (spleen was not removed)]. Organs measured had intrinsic fluorescence. The measured fluorescences are shown in Table 2. The gills, a priority organ in its contact with xenobiotics, showed the most significant accumulation of nanoparticles (Table 2). Mean concentrations of nanoparticles detected in the blood were 16.5 ± 0.7 ng/mg blood protein in male and 10.5 ± 2.2 ng/mg blood protein in female with 10 mg/L of ambient water. Fluorescence was detected from the blood, confirming that nanoparticles had entered through the gills. Nanoparticles that entered the bloodstream would reach the liver, gallbladder, and kidney. Also, nanoparticles would simultaneously enter the liver and gallbladder from intestinal adsorption after oral administration because the intestine showed a significantly high accumulation of nanoparticles. Surprisingly, nanoparticles were detected in brain and testis, although the *p-*values for these results were not significant (Table 2). The fluorescence intensity of the ovaries in exposed females was high compared with that in other organs, but the ovaries showed intrinsic fluorescence. There was no difference between the exposed and nonexposed data.

## Discussion

I used water-suspended fluorescent nanoparticles to investigate the distribution of nanoparticles in fish eggs and bodies. Particles 39.4–42,000 nm in diameter were adsorbed on the chorion of medaka eggs and accumulated in the oil droplets. Particles 474 nm in diameter showed the highest bioavailability to eggs (Figures 1 and 3), and 39.4-nm particles were confirmed to shift into the yolk and gallbladder along with embryonic development (Figure 2). Also, adult medaka were exposed to 39.4-nm nanoparticles at 10 mg/L, and these particles were detected at high levels in the gills and intestine (Table 2). I believe the nanoparticles pass through the membranes of the gills and/or intestine and enter the circulation. Fish gills are well known to be the main organs of xenobiotic uptake into the fish body from ambient water. Therefore, it is believed that most nanoparticles were taken up into organs via the gill-blood route. Jani et al. (1990) orally administered polystyrene nanoparticles to rats and showed the intestine–blood route for distribution of nanoparticles into organs. Uptake of nanoparticles via the intestine may contribute to distribution of nanoparticles in fish. Nanoparticles penetrated the blood–brain barrier to reach the brain, although the amounts of nanoparticles that reached the brain were low (Table 2). Oberdörster (2004) proposed that exposure of the brain of largemouth bass to C_60_ fullerenes could occur via olfactory neurons. Transport of nanomaterials to the brain via the olfactory neurons occurs in mammals (Oberdörster et al. 2004). However, in medaka the nanoparticles entered the circulation through the membranes of the gills and/or the intestine, and evidence of olfactory neuron migration of particles was not found.

When embryos were exposed to 39.4-nm nanoparticles at 1 mg/L, nanoparticles were rarely detected in the livers of posthatch larvae (Figure 2), and nanomaterials were distributed mainly in the yolk and gallbladder. The liver serves three main functions: *a*) uptake, metabolism, storage, and redistribution of nutrients and other endogenous molecules; *b*) metabolism of xenobiotics; and *c*) formation and excretion of bile (Hinton et al. 2001). Although there is little information on whether these three functions are already operable in posthatch larvae of medaka, posthatch larvae can at least take up yolk into the liver as nutrients via the left duct of Cuvier and the left hepatic vein during the yolk-adsorption period (Hinton et al. 2004). Therefore, it is possible that nanoparticles accumulated in the yolk will shift to the liver through the blood after the embryos hatch. In addition, nanoparticles shifted to the liver and intestine of larvae and were eliminated in the feces (posthatch day 2, data not shown), as was the case in adult rats (Yamago et al. 1995). Nanoparticles could not be detected in the spleen by fluorescence microscopy (Figure 5). The spleen is a vital organ in the immune system, producing antibodies in the form of lymphocytes and reabsorbing old blood by phagocytosis (Anderson 1992; Siwicki et al. 1990). Yamago et al. (1995) in a study of the distribution of water-miscible C_60_ fullerene in rats found that intravenously administered water-miscible fullerene was distributed mainly to the liver (91.7% of dose); some was distributed to the spleen (1.6% of dose) and other organs (Table 2). Nanoparticles were detected in the blood of ST II medaka at mean concentrations of 16.5 and 10.5 ng/mg blood protein in male and female, respectively, although they were not detectable in the blood by fluorescence microscopy because of their low concentrations. The concentration of fullerene distributed in the blood of rats is reported to be 0.57% of the dose (Yamago et al. 1995). The distribution of water-suspended nanoparticles was low in the blood and spleen compared with other organs. Nanoparticles may not pass through the cell membrane of the spleen. However, the more redox-active and smaller nanoparticles could penetrate organs by causing inflammation, then passing through the inflamed cell membranes and possibly causing a functional decline in the liver, spleen, and other organs. The distribution of nanoparticles in medaka was similar to that of radioactive fullerene in rats (Yamago et al. 1995), as both materials were distributed throughout body via blood flow, and therefore, the liver became a primary organ exposed to nanomaterials.

Lethal toxicity in medaka embryos exposed to 39.4-nm nanoparticles at 30 mg/L was observed (Table 1), although there was no lethal effect upon exposure to 1 mg/L (data not shown). Interestingly, the lethal effect increased proportionally with the salinity, and 100% complete lethality occurred at 5× and higher concentrated ERM solutions (Table 1). In addition, adsorption and accumulation of nanoparticles peaked with 15× ERM solution, then decreased with 20× and 30× ERM solutions (Figure 4). Simultaneously, nanoparticles aggregated in concentrated ERM solutions. In these respects, the adsorption, accumulation, and toxic effects of nanoparticles in medaka embryos must be related to salinity. Salinity may affect the bioavailability of nanoparticles to penetrate membranes. Sakaizumi (1980) reported a salinity-dependent lethal effect of methyl mercuric chloride on the hatchability of medaka embryos: exposure of embryos to methyl mercuric chloride at 100 μg/L in 0.1-to 1-mM NaCl solutions (5.8–58.4 mg/L) had 100% complete lethal effects, but there was no lethal effect at much lower NaCl concentrations (Sakaizumi 1980). Even if nanoparticles do aggregate in high-salinity solutions and become larger particles, particles in a certain range are adsorbed on, and accumulate in, medaka eggs (Figure 3). There is very limited information about how nanoparticles penetrate the egg chorion and accumulate in the yolk; furthermore, we have no data on the threshold of toxicity in aquatic organisms and environmental relevance of risk by nanomaterials.

Hardman (2006) in a recent review of the toxicity of QDs noted that the current literature reveals that assessing QD potential toxicity is not a simple matter: not all QDs are alike, and their toxicity depends on multiple physicochemical and environmental factors. This statement most likely applies not only to QDs but also to all nanoparticles. In this investigation I examined the distribution of nanoparticles in fish and embryos and demonstrated that nanoparticles are taken up into the medaka body from the ambient water and distributed throughout the body via the blood flow. I also showed that nanomaterials have salinity-dependent bioavailability and toxic effects. The biological activity of nanoparticles depends on physicochemical characteristics such as particle size, chemical composition, surface structure, solubility, shape, and aggregation (Nel et al. 2006). Nanotoxicology studies of gene, endocrine, and immune systems and reproduction in living organisms, as well as environmental studies of the fate and effects of nanomaterials, are needed to define the risks and benefits of nanomaterials applications. Until more is known about the environmental effects of nanomaterials, the release of manufactured nanomaterials into the environment must be avoided as far as possible.

## Figures and Tables

**Table 1:** Effects of ERM salinity on nanoparticle adsorption and accumulation by ST II eggs, nanoparticle aggregation in ERM solution, and embryo mortality.

		In the absence of fluorescent nanoparticle			In the presence of fluorescent nanoparticle		
ERM^a^	Osmotic pressure (mOsm/L)	Fluorescence^b^ of eggs	Optical density^c^ of solution	Mortality rate of embryos [24 hr (%)]	Fluorescence^b^ of eggs	Optical density^c^ of solution	Mortality rate of embryos [24 hr (%)]
1×	33.3	4.6	0.00	0.0	44.9	0.19	35.6
5×	167	4.6	0.00	0.0	74.4	0.33	97.8
7.5×	250	4.6	0.00	0.0	107	0.30	97.8
10×	333	4.6	0.00	0.0	144	0.37	100
15×	500	4.5	0.00	0.0	188	0.27	100
20×	666	4.6	0.00	0.0	79.7	0.62	100
30×	1,000	4.5	0.00	0.0	15.0	1.1	100

Fluorescent particles 39.4 nm in diameter (30 mg/L ERM) were used.

a 1× ERM was composed of 1 g NaCl, 0.03 g KCl, 0.04 g CaCl_2_ × 2H_2_O, and 0.163 g MgSO_4_ × 7H_2_O in 1 L ultrapure water and adjusted to pH 7.2 with 1.25% sodium bicarbonate solution.

b Fluorescence intensity at excitation wavelength 480 nm and emission wavelength 510 nm.

c Measured at 600 nm.

**Table 2:** Distribution of 39.4-nm fluorescent latex particles (10 mg/L ERM) in organs of ST II medaka after 7 days’ exposure, and comparison with rats.

Organs (no.)	Control^a^ (mean ± SE)	Exposure^a^ (mean ± SE)	*p*-Values^b^	Distribution of water-miscible C_60_ fullerene in rats^c^ (% total dosed radioactivity)
Brain (16)	28.2 ± 5.6	57.1 ± 8.7	0.080	0.57 ± 0.19
Gills (16)	28.3 ± 4.2	113 ± 10	0.000029	NA
Liver (16)	48.7 ± 6.2	93.9 ± 19	0.067	91.7 ± 8.0
Kidney (16)	62.6 ± 14	103 ± 16	0.068	1.0 ± 0.3
Gallbladder (16)	183 ± 30	246 ± 3.1	0.028	NM
Intestine (16)	25.8 ± 3.7	147 ± 20	0.00046	NM
Spleen	NM	NM	NA	1.6 ± 0.2
Lungs	NA	NA	NA	1.0 ± 0.3
Testis (8)	47.1 ± 18	112 ± 15	0.052	0.09 ± 0.01
Ovary (8)	118 ± 67	129 ± 36	0.44	NM

Abbreviations: NA, not available; NM, not measured.

a Fluorescence intensity at excitation wavelength 480 nm and emission 510 nm.

b ANOVA.

c Data from Yamago et al. (1995).
